# Prevalence, phenotype and inheritance of benign neutropenia in Arabs

**DOI:** 10.1186/1471-2326-9-3

**Published:** 2009-03-27

**Authors:** Srdjan Denic, Saad Showqi, Christoph Klein, Mohamed Takala, Nicollas Nagelkerke, Mukesh M Agarwal

**Affiliations:** 1 Department of Internal Medicine, Faculty of Medicine and Health Sciences, UAE University, Al Ain, UAE; 2 Hematology Laboratory, Department of Pathology, Al Ain Hospital, Al Ain, UAE; 3 Department of Pediatric Hematology/Oncology, Medical School Hannover, Hannover, Germany; 4 Staff Clinic, Al Ain Hospital, Al Ain, UAE; 5 Department of Community Medicine, Faculty of Medicine and Health Sciences, UAE University, Al Ain, UAE; 6 Department of Pathology, Faculty of Medicine and Health Sciences, UAE University, Al Ain, UAE

## Abstract

**Background:**

Benign neutropenia, i.e., neutropenia not associated with an increased risk of infection, may result in serious medical consequences when a 'standard' definition of neutropenia (absolute neutrophil count (ANC) < 1.5 × 10^9^cells/L) is universally applied to all races. The aims of this study were to determine the prevalence of benign neutropenia among healthy Arabs and evaluate its mode of inheritance.

**Methods:**

ANCs were studied prospectively amongst a healthy indigenous population (n = 1032) from the United Arab Emirates undergoing a nation-wide sickle-cell and thalassemia screening program. The mean neutrophil count and the prevalence of benign neutropenia were compared by age, sex and amongst various tribes.

**Results:**

The mean neutrophil count (× 10^9^cells/L) was 3.3 (range 0.95–7.6). Benign neutropenia was present in 110 (10.7%) subjects of whom 24 (2.3%) individuals had moderate neutropenia (ANC 0.5 – 1.0 × 10^9 ^cells/L). In the 22 tribe-family groups, the prevalence of benign neutropenia varied between 0% and 38%. Benign neutropenia showed no difference in the frequency amongst the sexes (p = 0.23) and it was independent of age (Spearman's rho = 0.05, p = 0.13). The age-related mean neutrophil count was the lowest in Arabs when compared with other ethnic groups (Blacks, Europeans and Mexicans). The inheritance of benign neutropenia was consistent with an autosomal dominant pattern; however, the diversity of observed phenotypes suggested the presence of more than one genetic variant for this trait.

**Conclusion:**

Arabs have a high prevalence of benign neutropenia that may be inherited as an autosomal dominant trait.

## Background

Neutropenia is defined as an absolute neutrophil count (ANC) of less than 1.5 × 10^9 ^cells/L. During the second half of the 20^th ^century, this cutoff value was derived from clinical trials which were evaluating the myelosuppressive effects of chemotherapy. Most patients recruited for these seminal studies were of European origin; subsequently, it was realized that this 'standard' caused an excessive number of healthy individuals of African descent to be mislabeled as neutropenic. The neutropenia that is not associated with an increased risk of infections has been called benign neutropenia (BN). Using this description, BN has been reported in up to 30% of people of African origin [1]. Recently, the prevalence of neutropenia in American Blacks was found to be 4.4% compared to 0.8% in Whites [2]. Acquired chronic idiopathic neutropenia, which uses a higher cutoff for the neutrophil count and is related to inflammation, should be distinguished from ethnic BN [3,4].

This blanket definition of neutropenia may have serious implications in clinical practice. In patients on myelosuppressive therapy, the infectious complications of neutropenia are prevented by reducing the drug dose that is dependent on the neutrophil nadir. Thus, BN in healthy Blacks may cause an inappropriate reduction of the dose of the chemotherapeutic agent with serious consequences. It is possible that the poorer survival of black women with breast cancer may be partly due to this fact [5,6]. The anti-schizophrenia drug clozapine, which often causes neutropenia, is more likely to be discontinued in Blacks than Whites [7]. Similarly, the drug is less often initiated in Blacks due to a fear of the dreaded agranulocytosis [8]. There are other potential problems in subjects with BN which may occur: an unnecessary use of antibiotics for febrile non-bacterial illnesses and inappropriate hematological investigations. As in Blacks with BN, other ethnic groups known to have a 'low' neutrophil count (e.g., Yemeni and Ethiopian Jews) may be exposed to the same risks [9,10]. In Arabs, the reference range for neutrophil count has not been established; therefore, the prevalence of BN is unknown. Some reports suggest that Arabs also may have a lower neutrophil count when compared to people of European descent [10-12]. Arabs comprise of approximately 300 million people from 29 countries; they often live in tribal groups and practice endogamy, a custom that could alter the prevalence of genetic conditions like BN. Therefore, establishing reference values of normal neutrophil counts in healthy Arabs is very important to prevent any potential harm arising from an inappropriate reference range. The aims of this study were a) to determine the prevalence of BN (i.e., ANC < 1.5 × 10^9 ^cells/L) in an unbiased sample of healthy Arabs, b) to analyze dynamics of neutrophil count in subjects with BN, and c) to establish the mode of inheritance in selected families with BN.

## Methods

### Study population

Blood samples were drawn (once) over a six-month period (March, 2007 – August, 2007) as part of a nation-wide population-screening program for thalassemia and hemoglobin S. This screening center is located in the city of Al Ain, United Arab Emirates (UAE), which is on the border with Oman and in close proximity to Saudi Arabia. This screening program is mandated and fully funded by the government. It is restricted to the citizens of the UAE who intend to marry; therefore, the expatriate population was excluded from this study. In the local UAE population, marriages are arranged, often consanguineous, i.e., between cousins, and cannot proceed without a judge issuing a wedding-license after verifying the screening center report. There were no exclusion criteria, but data was missing in 47 (4.5%) individuals, who could not be used; the remaining 1032 consecutive healthy men and women were included in the final analysis.

The population is strictly tribal, with a total of 67 tribes in the country (as per 1968 population census) [13]. The tribe, subtribe, and family group allegiance of 78% of subjects was successfully determined from their last names. Members of at least four of the tribes in this study inhabit territories of other four Gulf countries because until very recently there were no barriers to the movement of people in this region.

A second cohort comprising of seven subjects with BN were from hematology clinic; they were used in analysis of the phenotype and the pattern of inheritance of neutropenia trait. In these subjects, the diagnosis of BN was based on 1) the negative history of use of medications known to cause neutropenia, 2) absence of chronic diseases associated with neutropenia, 3) absence of infection, 4) normal physical examination. Moreover, the laboratory tests confirmed the absence of anemia, thrombocytopenia and anti-nuclear antibodies, normal B_12 _and folate levels, normal bone marrow examination (in three of seven subjects) and documented neutropenia in at least one family member (in six of seven subjects). In all seven subjects, neutrophil counts were obtained from their medical records. Additionally, in two brothers with multiple ANC < 0.5 × 10^9 ^cells/L but without recurrent infections, we excluded a mild form of congenital neutropenia by excluding the presence of the mutations at ELA2 and HAX1 locus [14,15].

### Ethical Approval

This study was approved by Al Ain Medical District Human Research Ethics Committee (Protocol # 07/123).

### Laboratory Methods

The complete blood count (CBC) was performed once on every individual after the blood was collected in a tube containing EDTA and analyzed using the Cell-Dyn Sapphire (Abbot Diagnostics, USA) analyzer. The hospital laboratory subscribes to an overseas external quality control from the United Kingdom (UK National External Quality Assessment Scheme). The standard deviation index of leukocyte counts varied from -0.77 to 1.33 from the target mean value during the study period. Hence, the laboratory met the analytical standards for CBC.

### Statistical Methods

Data were logged into a computer database and analyzed using SPSS ver.15 for Windows (SPSS Inc., Chicago, Ill., USA). Standard descriptive and analytical statistical methods, such as histograms, scatter plots, linear regression and independent samples t-tests, Spearman's and Pearson's correlation coefficient were used. In addition, the ranges of cell counts were determined by taking the mean ± 2 SD on a Box-Cox (power) transformed scale and back transformation of the normal range limits. The figures were plotted using the SPSS and Excel (Microsoft). The level of statistical significance was 0.05.

## Results

**Figure 1:**
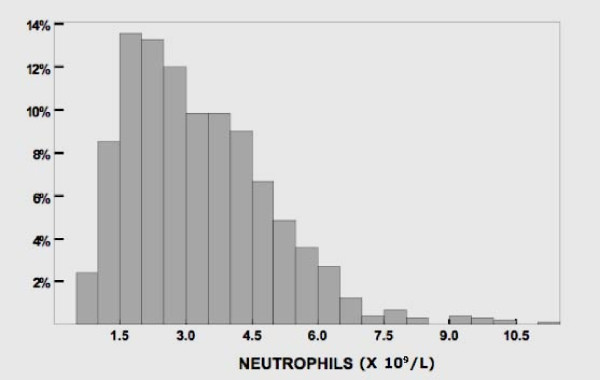
**Neutrophil count (n = 1032)**.

**Figure 2:**
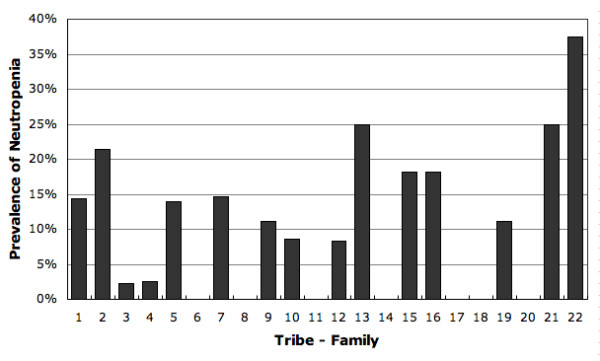
**Prevalence of neutropenia in 22 tribe-family groups**.

**Figure 3:**
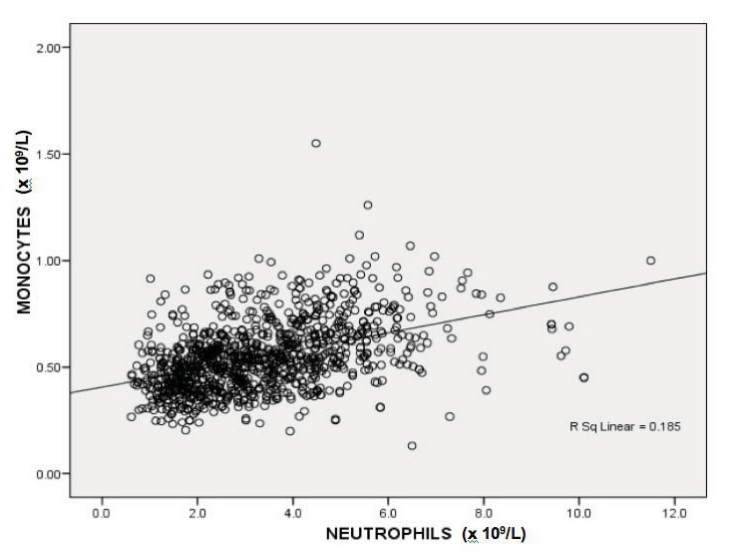
**Correlation of monocyte and neutrophil count in 1032 subjects**.

The characteristics of 1032 subjects, their differential leucocyte count and the prevalence of neutropenia are shown in Table 1; there was no significant statistical difference in any of these parameters between the sexes and age groups. All 24 subjects with ANC<1.0 × 10^9 ^had normal hemoglobin and platelet counts; therefore, hematological disorders causing the marked neutropenia was unlikely. The histogram of neutrophil counts is shown in Figure 1. In 22 largest population groups (defined by tribe, subtribe or family allegiance), the prevalence of neutropenia varied between 0% and 38% (Fig 2). A non-Arab tribe, which immigrated to Arabian Peninsula a few centuries ago (group 6 in Fig 2), had no neutropenic subjects as well as six Arab tribe-family groups. In our study cohort, the neutrophil and monocyte counts were positively correlated (Pearson's *r *= 0.43) (Fig 3). The subjects with neutropenia had a significantly lower monocyte counts than subjects without neutropenia (p < 0.001).

**Table 1:** Study subjects, mean leukocyte differential values* and prevalence of neutropenia.

	Number
All	1032
Males	517
Females	515
Mean Age & range, yrs	
All	24.4 (11–69)
Males	26.1 (16–69)
Females	23.0 (11–44)
Leucocytes (× 10^9^/L)	6.7 (3.5–11.5)
Neutrophils (× 10^9^/L)	3.3 (0.95–7.6)
Lymphocytes (× 10^9^/L)	2.6 (1.4–4.4)
Monocytes (× 10^9^/L)	0.5 (0.3–0.9)
Eosinophiles (× 10^9^/L)	0.17 (0.02–0.5)
Basophiles (× 10^9^/L)	0.03 (0.01–0.08)

ANC < 1.5 × 10^9^/L	10.7%
ANC < 1.0 × 10^9^/L	2.3%

* Mean ± 2 SD

ANC, absolute neutrophil count

**Figure 4:**
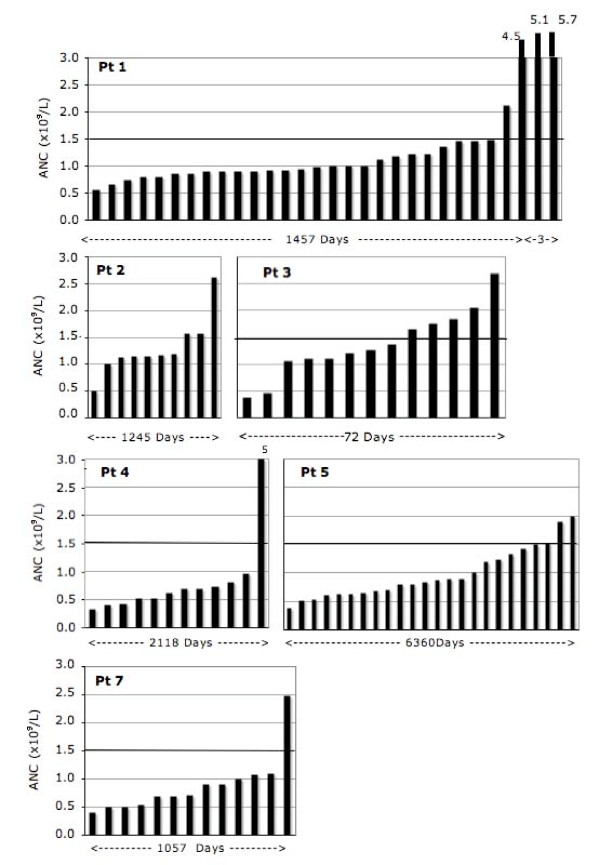
**Dynamics of the neutrophil count in six patients (Pt) with benign neutropenia sorted in ascending order**. Period on x-axis indicates duration of observation.

**Figure 5:**
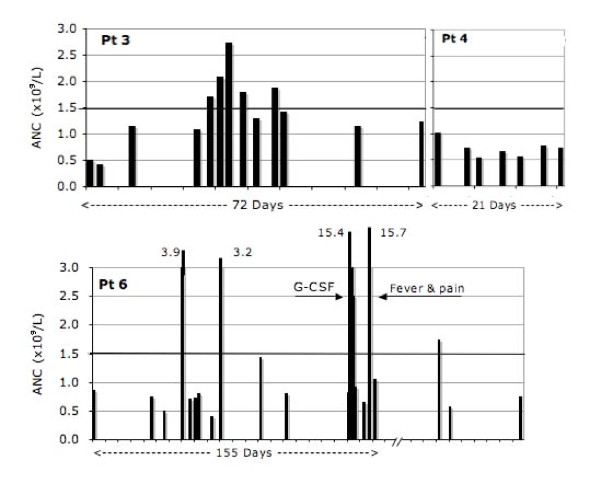
**Dynamics of the neutrophil count in three patients (Pt) with benign neutropenia in actual time sequence**. Ticks on x-axis are weeks.

**Figure 6:**
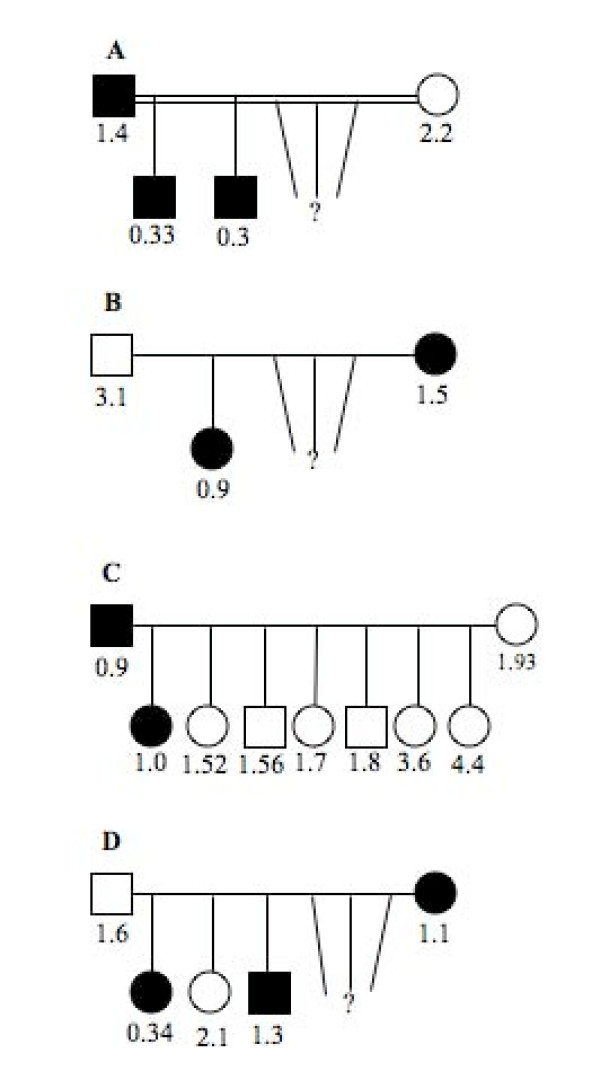
**Pedigree of families with benign neutropenia**. The numbers are the lowest absolute neutrophil counts recorded. Filled square and circle indicates the presence of neutropenic trait in male and female, respectively.

In seven subjects with BN from the hematology clinic, ANC was analyzed in more detail. In contrast to 1032 study subjects from the general population among whom none had an ANC < 0.5 × 10^9 ^cells/L, six of seven clinic subjects had "agranulocytic" neutropenia at least once; all seven, however, had ANC in the normal range once or more often (Fig 4 and 5). In order to better characterize the phenotype of BN in these patients, examination of ANC (sorted in ascending order in Figure 4) suggested the possibility of a mild neutropenia (in patients 2 and 3), more profound neutropenia (in patients 4 and 7) and possibly a cycling neutropenia (in patients 3 and 6 in Figure 5). Finally, to determine the Mendelian mode of inheritance, the pedigrees of the four families with BN were plotted in Figure 6. They are all consistent with autosomal dominant mode of inheritance of BN.

## Discussion

### Prevalence of neutropenia

**Figure 7:**
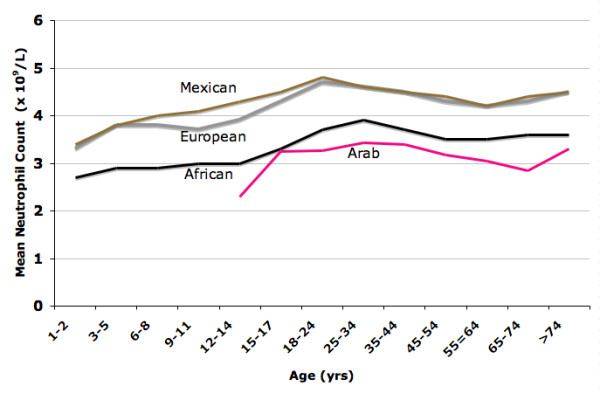
**Mean neutrophil count (MNC) in selected ethnic groups by age**. MNC for Mexicans, Europeans and Africans is from reference 2.

There is a paucity of data on neutropenia amongst Arabs. In this study, the prevalence of BN (ANC < 1.5 × 10^9 ^cell/L) and marked neutropenia (ANC 0.5 – 1.0 × 10^9 ^cell/L) in Arabs is 10.7% and 2.3% respectively. As the subjects with BN periodically have ANC in the normal range (Fig 4 and 5), the observed prevalence of BN in the studied population of Arabs may well be an underestimate. The mean neutrophil count in Arabs is 3.3 × 10^9^/L (range, 0.95–7.6), without any significant difference between the sexes. As ethnicity is known to affect neutrophil count, we compared the mean neutrophil count of the Arab subjects in this study, of mainly young adults, with those previously reported in American Blacks, Whites and Mexicans [2]. In Arabs, ANC is lower than the mean neutrophil values earlier reported for younger (age <18 years) and older (age ≥ 18 years) American Mexicans, Whites and older Blacks [2], as is shown by plotting neutrophil counts by age for these ethnic groups (Fig 7).

In this study, the neutrophil count correlated with the monocyte count (r = 0.43). This has not been previously reported and is in contrast to patients with congenital neutropenia who often show a relative increase in monocytes counts. The association of neutropenia and monocytopenia may imply that subjects with BN may also be monocytopenic. This may give a possible clue to its origin. For example, in cyclic congenital neutropenia, synchronized oscillation of neutrophils and monocytes may be related to a back loop inhibition from neutrophils to their common precursor cell [16]. Therefore, a concordant decrease of neutrophils and monocytes may suggest that the mechanism may involve a common progenitor cell.

In this study, there was no sex bias as there were equal numbers of healthy males and females. A study of a benign genetic condition in a population that is shortly going to reproduce is most useful for estimating its prevalence. Similarly, there was no selection bias as the blood tests in the study were mandatory without any cost to the subjects; further, we identified 73 different tribes, subtribes and family groups. However, the parameters which may increase neutrophil count, e.g., smoking were not controlled for in the study.

Our findings confirm and extend previous reports on neutropenia in the Arab Peninsula. BN has been previously reported in Jordanians and Bedouin Arabs (from the north of Arabian Peninsula), Kuwaiti citizens (in central part of Peninsula) as well as among Yemeni and Ethiopian Jews (many residing in southern part of the Peninsula), and amongst Arab tribe of Sudanese origin residing in the Middle East [9-12]. In addition, four of the tribes with neutropenia in our study are present in four other Arab Gulf countries. This suggests that a high prevalence of BN is likely to be present in most populations in the Middle East and North Africa.

### Phenotype of benign neutropenia

In the second part of the study, to determine the phenotype of BN, we analyzed seven subjects from the hematology clinic. We sorted (in ascending order) all available ANC of these patients, which suggested three possible phenotypes of BN: mild, profound and cycling types of neutropenia. This classification is tentative due to a limited number and irregularity of data points as well as cycling of neutrophil counts in normal subjects and unavoidable background noise [17,18]. Nonetheless, in several subjects the impressive part was the benign nature of the condition (absence of infections) even when ANC was < 0.5 × 10^9 ^cells/L. In one family, two healthy sons had frequently ANC < 0.5 × 10^9^cells/L and a mild form of congenital neutropenia was exclude with the negative tests for mutation at ELA2 and HAX1 gene locus. In contrast to the absence of severe neutropenia in 1032 screened individuals, its presence in six clinic patients could be explained by a larger number of measurements of ANC, selection bias, possible effects of less frequent alleles or homozygosity (codominant inheritance), alone or in combination.

The results of this study imply that Arabs have two healthy subpopulations with different ANCs. How big these subgroups are is unclear because of a high variability of neutrophil count and a need for multiple sampling in order to properly classify all the subjects. Nonetheless, in clinical practice this requires two cutoff values for neutropenia, one of 1.0 × 10^9 ^cells/L and perhaps less for neutropenic population and another of 1.5 × 10^9 ^cells/L for non-neutropenic subpopulation. The use of a single value of ANC for defining neutropenia in such a population would also endanger a healthy subpopulation with a higher neutrophil count; it would be put at risk of over-treatment with chemotherapy, under-investigation of neutropenia or under-utilization of antibiotics for infections. Therefore, identification of healthy subjects with lower neutrophil count through screening may be required in this population.

### Inheritance and population genetics of benign neutropenia

Earlier report suggested that BN in an Arab tribe is inherited as autosomal dominant trait. In that report, however, ANC < 2.0 × 10^9^/L was used as a definition of neutropenia [10]. Our analysis of four families using ANC < 1.5 × 10^9^/L as a cutoff value for neutropenia supports autosomal dominant mode as a likely pattern of Mendelian inheritance (Fig 6). However, observation of a profound neutropenia in several subjects (Figs 4 and 5) suggests possibility of the existence of a more than one mutation for BN in this population.

An indication that BN may be inherited as an "ancient" and widely spread trait comes from the presence of neutropenia in diverse Arab tribes (Fig 2). As endogamy was strictly enforced and inter-tribe marriages were infrequent in the past, the presence of neutropenia in many tribes suggests that its presumed gene was present long before tribal partitioning. In the more recent past, a more intense mixing of people than commonly presumed is evidenced by both historical analysis of migrations and high diversity of alpha-thalassemia mutations in these populations [13,19]. The observed variations in the prevalence of neutropenia, however, could be due to a sample size, founder effect, and different rates of expansion and mixing of populations. The effect of widespread consanguinity (increased homozygosity due to inbreeding) on BN could be determined only after a better phenotype-genotype correlation is established. Finally, that BN may be ubiquitous in Arabs is suggested by a high prevalence of neutropenia in people of African origin; being neighbors they could have exchanged genes during their long and common history. However, the purpose, adaptive value and evolutionary origins of BN still need to be addressed.

### Malaria hypothesis

It may be possible that the inter-ethnic differences in neutrophil counts may be the consequence of human adaptation to malaria, similar to the thalassemias and hemoglobin S. We base this speculation on three epidemiological observations. First, there is a superimposition of the distribution of *Plasmodium falciparum *and the geographic distribution of populations with low neutrophil counts. Second, there is an inverse correlation between the intensity of exposure to *Plasmodium falciparum *and the mean neutrophil count in the same population. Historically (before Columbus), Mexicans were never exposed to malaria and have the highest mean neutrophil count (Fig 7) and the lowest prevalence of neutropenia (0.4%) [2]. In contrast, the Arabs and Africans have a long history of endemic malaria and both have low mean neutrophil count and a high prevalence of neutropenia of 10.7% and 4.4%, respectively [1,2]. Similarly, ANCs are lower in Yemeni and Ethiopian Jews, who both reside in regions with malaria and have a high frequency of neutropenia [9,10]. Historically, Europeans had a relatively mild exposure to *P. falciparum *and their mean neutrophil count and the prevalence of neutropenia (0.8%) are also low. A third observation in support of malaria hypothesis is a direct correlation between the age-specific mortality from malaria and the age-specific prevalence of BN. Mortality from malaria is highest in the first five years of life when the prevalence of neutropenia appears to be highest [2]. The mechanism by which a low neutrophil count could provide a survival advantage in malarial infections is unclear. Neutrophils are charged with multiple toxic molecules which could direct destructive processes in the brain and lungs which are the most common causes of deaths due *P. falciparum *[20]. Thus, a lower neutrophil and monocyte count found in subjects with BN may be associated with fewer deaths from malaria by causing a less destructive tissue reaction.

## Conclusion

The mean neutrophil count in Arabs is lower than in individuals of European origin. This is the consequence of a high prevalence of BN, a genetic condition which may have an autosomal dominant mode of transmission. These findings may be extrapolated to other Arab societies; we contend that a high prevalence of BN is the consequence of adaptation to endemic malaria. BN may result in several adverse consequences if not classified as 'benign'; therefore, using a lower neutrophil count to define neutropenia would prevent many of these undesirable effects. However, this may cause potential problems in Arab subpopulations with an inherently higher neutrophil count. Thus, a potential solution to this problem is screening the population for neutropenia. Progress in high throughput sequencing technology, and a more refined and extended pedigree analysis may allow the identification of genetic alterations responsible for the differing neutrophil counts in the Arab population. This may eventually reveal novel pathways controlling homeostasis of neutrophils and facilitate development of clinical guidelines for dealing with BN.

## Competing interests

The authors declare that they have no competing interests.

## Authors' contributions

SD conceived, designed, and organized the study; furthermore, analyzed the results and wrote the manuscript. SS participated in the design of the study, performed the test and data collection. SK carried out the genetic analysis and helped with the draft of the manuscript. MT assisted in coordination of the study. NN performed the statistical analysis and helped with the draft of the manuscript. MMA participated in the presentation of the study and rewrote portions of the manuscript. All authors read and approved the final manuscript.

## Pre-publication history

The pre-publication history for this paper can be accessed here:


